# Evaluating the association between job stress and presenteeism among nurses: the mediating role of emotional exhaustion and the moderating effect of leisure crafting

**DOI:** 10.3389/fpubh.2026.1719915

**Published:** 2026-01-26

**Authors:** Cheng Zhang, Jimin Feng, Dongwen Li, Hongxu Wang

**Affiliations:** 1 School of Nursing, Chengdu Medical College, Chengdu, China; 2 Department of Stomatology, General Hospital of the Western Theater of the Chinese People’s Liberation Army, Chengdu, China; 3 School of Nursing, North Sichuan Medical College, Nanchong, China

**Keywords:** emotional exhaustion, job stress, leisure crafting, nurses, presenteeism

## Abstract

**Background:**

Exploring the relationship between job stress and presenteeism is essential for safeguarding nurses’ well-being and patient safety. To further clarify the mechanisms underlying this relationship, this study applied the Conservation of Resources theory (COR) and the Job Demands-Resources (JD-R) model to examine the mediating role of emotional exhaustion between job stress and presenteeism, with particular emphasis on the potential moderating role of leisure crafting in this process.

**Methods:**

A cross-sectional survey was conducted from April to June 2025, involving 458 nurses recruited via convenience sampling from three tertiary Grade-A general hospitals in Sichuan Province, China. Data were collected via scales measuring job stress, emotional exhaustion, leisure crafting, and presenteeism. Mediation and moderation analyses were conducted using Hayes’ PROCESS macro (Models 4 and 7) in SPSS 26.0 with 5,000 bootstrap samples.

**Results:**

The results showed that job stress was positively associated with presenteeism among nurses. Emotional exhaustion partially mediated this relationship. Higher job stress was linked to greater emotional exhaustion, which in turn was associated with higher levels of presenteeism. Leisure crafting significantly moderated the link between job stress and emotional exhaustion and the association was weaker among nurses with higher levels of leisure crafting. The moderated mediation effect was significant, suggesting that leisure crafting buffered the indirect effect of job stress on presenteeism via emotional exhaustion.

**Conclusion:**

Emotional exhaustion serves as a key psychological mechanism through which job stress translates into presenteeism among nurses. Job stress may exacerbate nurses’ susceptibility to suboptimal health by increasing emotional exhaustion, thereby accelerating their progression along the continuum of occupational health deterioration. Nurses experiencing suboptimal health are more likely to engage in presenteeism. Leisure crafting serves as a valuable resource for alleviating emotional exhaustion and mitigating presenteeism among nurses under stress. These results enhance the understanding of the mechanisms underlying nurse presenteeism and offer practical implications for developing supportive interventions in nursing management.

## 1 Introduction

Presenteeism refers to the act of continuing to work despite experiencing physical or psychological discomfort or illness, typically manifested as reduced productivity and inefficient work performance (1). Due to heavy workloads and elevated health risks, nurses are considered a high-risk group for presenteeism, with an incidence approximately three to four times higher than that of other occupational groups (2). Long-term presenteeism exacerbates nurses’ physical and psychological fatigue, which exacerbates health impairments, including severe discomfort and burnout (3), and also compromises the quality of nursing care and jeopardizes patient safety (4). It also leads to reduced work engagement and performance, resulting in significant economic costs (5). Therefore, it is of great theoretical and practical significance to investigate the underlying mechanisms of nurse presenteeism and to identify effective intervention strategies.

In recent years, research on nurse presenteeism has increased. However, most existing studies have been conducted in Western cultural settings, with relatively limited empirical evidence from Eastern cultural backgrounds (6). In China, rapid socio-economic and healthcare reforms have placed increasing demands on nurses, contributing to high levels of job stress. Factors such as workforce shortages, excessive workloads, and elevated responsibility for patient outcomes, all increase the likelihood of working while sick (7). Additionally, influenced by collectivist cultural norms, traditional Chinese values place strong emphasis on dedication and work responsibility, which may further reinforce nurses’ tendencies toward presenteeism (8, 9). Previous studies have identified that multiple workplace stressors, such as workload, time pressure, strained interpersonal relationships, and patient care demands, are significantly associated with increased presenteeism among nurses (7, 10). More significantly, these daily work stressors have a profound and lasting relationship with health impairment (11). Prolonged exposure to high job demands may progressively lead nurses into a suboptimal health state that represents an intermediate condition between full health and overt disease (12). This state is typified by chronic fatigue, emotional exhaustion, and reduced vitality (13), which may manifest as presenteeism (14). Nevertheless, the mechanisms underlying the relationship between job stress and presenteeism remain insufficiently understood, particularly due to a lack of systematic exploration regarding the pathways of job stress and potential moderating factors (15, 16). This gap hampers the formulation of robust theoretical models and practical intervention strategies.

Drawing on the health impairment process of the Job Demands-Resources (JD-R) model, emotional exhaustion not only represents resource depletion, but also potentially indicates the onset of health deterioration in nurses under stress (17, 18), and may serve as a mediating mechanism linking job stress to presenteeism. Additionally, informed by Conservation of Resources (COR) theory, we introduced leisure crafting as a proactive coping mechanism by which individuals acquire resources during leisure time to replenish resource depletion from job stress, thereby mitigating emotional exhaustion (19, 20). As a form of proactive resource compensation, leisure crafting may buffer the indirect effect of job stress on nurse presenteeism via emotional exhaustion.

In summary, this study developed a moderated mediation model to systematically elucidate the underlying mechanisms of nurse presenteeism. Drawing upon the JD-R model, we examined emotional exhaustion as a mediator linking job stress to presenteeism. We also investigated the moderating role of leisure crafting, which may buffer the adverse association of job stress with emotional exhaustion by providing physical and psychological resources. This study aims to deepen the theoretical understanding of nurse presenteeism and provide reliable empirical evidence and practical guidance for promoting nurses’ physical and psychological well-being.

## 2 Theoretical framework

**Figure 1:**
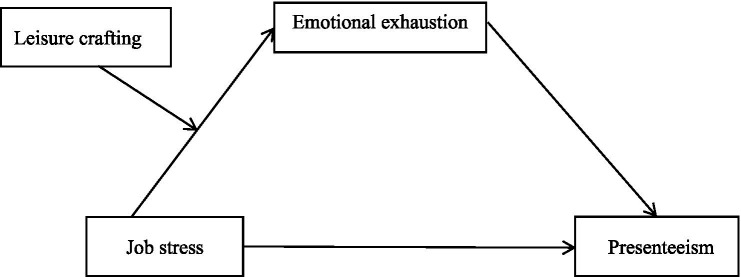
The proposed conceptual model.

This study is based on the JD-R model (18) and Conservation of Resources (COR) theory (20), which together provide a theoretical framework for understanding the relationship between job stress and presenteeism among nurses (see Figure 1). The JD-R model states that each occupation has specific job demands and job resources, and it consists of the motivational process and the health impairment process (12, 21). Excessive work demands deplete employees’ physical and mental resources, leading to stress and health issues. The COR theory posits that individuals are inherently motivated to acquire, retain, and protect valued resources. When resources are threatened or lost, individuals experience stress responses and attempt to prevent further depletion (20). Moreover, the theory emphasizes that when individuals experience resource insufficiency or loss, they may adopt resource substitution strategies by drawing on resources from one domain, such as leisure or family, to compensate for shortages in another domain, such as work, thereby enhancing their ability to cope with stress (22).

## 3 Literature review and research hypotheses

### 3.1 Job stress and presenteeism

Job stress refers to the psychological and physiological strain resulting from a mismatch between job demands and an individual’s abilities, resources, or needs (23). Nurses are frequently exposed to various stressors, including excessive workloads, time pressure, complex care environments, diverse patient conditions, and interpersonal demands (24). Persistent job stress can lead nurses to experience a range of physical and psychological health problems, including sleep disturbances, chronic fatigue, anxiety, and emotional exhaustion, and may further impair their work performance (25, 26). Job stress is recognized as a key antecedent of presenteeism among nurses (17, 27). From the perspective of COR theory, high-pressure nursing environments continuously deplete or threaten nurses’ core resources, such as health, time, and a sense of control (20). When nurses perceive potential resource loss, they activate resource conservation strategies to minimize further depletion (28). For nurses, taking sick leave may result in additional resource loss, including reduced income, disrupted schedules, increased burden on colleagues, or harm to professional reputation (29, 30). Under resource-threatening conditions, presenteeism may be adopted as a short-term strategy to conserve resources by maintaining attendance, especially in contexts of intense job demands and strict performance evaluations (31). However, such short-term conservation efforts may trigger a resource loss spiral, leading to further health deterioration and decreased work effectiveness among nurses (20, 32). Thus, we hypothesize that:

*H1*: Job stress is positively associated with presenteeism.

### 3.2 The mediating role of emotional exhaustion

Emotional exhaustion is defined as a state of extreme depletion of emotional and physiological resources often observed in contexts of prolonged job stress, with typical manifestations including continuous fatigue, emotional overextension, and lack of sufficient energy and ability to cope with daily job demands (33). Previous research indicated that emotional exhaustion is reciprocally related to chronic job stress (34). Emotional exhaustion can heighten individuals’ sensitivity to job stress and amplify their stress responses (35). When individuals are highly emotionally exhausted, their ability to manage routine work demands is reduced, making even ordinary tasks feel overwhelming and increasing perceived stress levels. Sustained exposure to demanding work conditions progressively drains individuals’ energetic resources, thereby exacerbating emotional exhaustion (36). Emotional exhaustion represents a lack of resources and energy, and it has a significant association with individual health and job performance (37, 38). A longitudinal study found that emotional exhaustion significantly predicted presenteeism in the following year (39). Viewed within a broader continuum of occupational health deterioration, emotional exhaustion represents more than a mere depletion of resources and energy (18). It is further recognized as a core symptom of suboptimal health status, signaling an early impairment of both physical and psychological health (13). When individuals are in this latent state of health impairment, they often find it difficult to maintain optimal work efficiency, which may manifest as presenteeism at work (14).

Furthermore, previous studies have shown that burnout mediates the relationship between job stress and presenteeism among healthcare workers (17). Emotional exhaustion is a key dimension of burnout, and whether it mediates the relationship between job stress and presenteeism among nurses requires further investigation. According to the health impairment process of the JD-R model, common job stressors for nurses are classified as job demands (13). Long-term excessive job demands deplete nurses’ physical and psychological resources, manifesting as a suboptimal health state characterized by emotional exhaustion and severe fatigue. If this condition persists, it may significantly impair nurses’ job performance (40). Therefore, on the one hand, long-term high job stress damages nurses’ physical and psychological resources, leading to emotional exhaustion. On the other hand, nurses in a prolonged state of emotional exhaustion may be unable to effectively engage in work due to severe energy depletion, resulting in presenteeism with reduced work efficiency despite physical attendance (41). Consistent with the JD-R model, Uslukaya and Demirtaş found that emotional exhaustion partially mediated the relationship between job demands and presenteeism (37). Thus, we hypothesize that:

*H2*: Emotional exhaustion plays a mediating role between job stress and presenteeism.

### 3.3 The moderating role of leisure crafting

Leisure crafting is a proactive behavior in which individuals intentionally engage in leisure activities to fulfill needs related to goal setting, interpersonal relationships, learning, and personal development (42). High levels of leisure crafting have been shown to enhance personal self-worth and subjective well-being (43), improve nurses’ work efficiency and engagement (44), and ultimately promote care quality and patient safety (45). According to COR theory, when individuals experience depletion or insufficiency of job-related resources, they tend to adopt “resource substitution” strategies by acquiring resources from non-work domains (e.g., family or leisure) to compensate for deficits in the work domain (22). In other words, when job stress consumes substantial personal resources, individuals may replenish them by acquiring resources during leisure time. Leisure crafting, in this context, serves as a key means of acquiring and restoring resources (46). For example, individuals can enhance their physical resources through active leisure (e.g., exercise) (47), and restore psychological resources through social interaction, skill development, or meaningful hobbies, gaining a sense of value, belonging, and fulfillment (48). Accordingly, leisure crafting may function as a cross-domain compensatory strategy that buffers the resource depletion associated with job stress and thereby alleviates emotional exhaustion (20). Previous studies have demonstrated a negative association between leisure crafting and emotional exhaustion, particularly in high-stress work environments (49, 50). A daily diary study by Abdel Hadi et al. further confirmed that leisure crafting not only reduces emotional exhaustion but also attenuates the adverse effect of high job demands on emotional depletion (51). Thus, leisure crafting may moderate the relationship between job stress and emotional exhaustion by acting as a protective factor. Specifically, for nurses who actively engage in leisure crafting, the negative impact of job stress on emotional exhaustion may be attenuated, which in turn may reduce the likelihood of presenteeism. Thus, we hypothesize that:

*H3*: Leisure crafting moderates the relationship between job stress and emotional exhaustion.

*H4*: Leisure crafting moderates the indirect effect of job stress on presenteeism through emotional exhaustion.

The contributions of this study are mainly reflected in the following three aspects: First, it integrates the JD-R model and COR theory to systematically explain how job stress is linked to presenteeism among nurses, both directly and indirectly via emotional exhaustion, thereby enriching theoretical understanding in the nursing field. Second, by introducing leisure crafting as a novel moderator, the study examines its buffering role between job stress and emotional exhaustion, thus expanding its application in the context of nurse occupational health research. Third, the findings provide targeted recommendations for nursing managers and policymakers, such as promoting leisure crafting and alleviating emotional exhaustion, offering practical guidance for reducing presenteeism and enhancing nurse well-being and care quality.

## 4 Methods

### 4.1 Participants

This cross-sectional study recruited participants from three tertiary Grade-A general hospitals in Sichuan Province, China, using a convenience sampling method, with data collected through an online questionnaire survey from April to June 2025. Questionnaires were distributed to hospital nurses after obtaining consent from the nursing departments of the hospitals. The inclusion criteria were as follows: (1) obtained a vocational qualification certificate from the People’s Republic of China; (2) at least 1 year of clinical nursing experience; (3) received informed consent and offered voluntary participation in the study. The exclusion criteria were as follows: (1) nurses who were off-duty due to leave, external study, or other reasons; (2) informal employees like training nurses and rotation nurses; (3) participants who provided incomplete questionnaires.

### 4.2 Sample size

According to the rough sample size estimation method (52), the sample size should be at least 5 to 10 times the number of variables. This study involved a total of 16 variables (7 sociodemographic and 9 scale-related dimensions), and considering 20% invalid questionnaires, the required sample size is approximately 100 to 200 cases. In this study, a total of 475 questionnaires were initially collected, and after excluding 17 questionnaires that did not meet the criteria, 458 valid questionnaires were retained, yielding a valid response rate of 96.42%.

### 4.3 Measurements

#### 4.3.1 Demographic characteristics

This study used a self-designed demographic questionnaire, which included 7 variables: nurses’ gender, age, marital status, educational level, professional title, self-rated health status, and years of work experience.

#### 4.3.2 Nurses’ job stressor scale

Developed by Chinese scholar Li et al. (53), it is used to analyze the job stress status of nurses. It includes five dimensions with a total of 35 items: professional and career issues, workload and time pressure, work environment and resources, patient care and interaction, and interpersonal relationships and management problems. A 4-point Likert scale is used for scoring, ranging from “strongly disagree” to “strongly agree,” with scores from 1 to 4. The total score ranges from 35 to 140, with higher scores indicating greater job stress. In this study, the Cronbach’s *α* of the scale was 0.974.

#### 4.3.3 Emotional exhaustion scale

Emotional exhaustion was assessed using the Emotional Exhaustion Scale (54). This scale consists of 5 items and uses a 7-point Likert scale (0 = strongly disagree, 6 = strongly agree), with a total score ranging from 0 to 30. Higher scores indicate a higher level of emotional exhaustion. In this study, the Cronbach’s *α* of the scale was 0.857.

#### 4.3.4 Leisure crafting scale

Originally developed by Petrou and Bakker (42), the scale was cross-culturally adapted into Chinese by Guo et al. (55) and renamed the Chinese version of Leisure Crafting Scale (C-LCS) to assess participants’ leisure crafting levels. The C-LCS is a unidimensional scale comprising 9 items. Each item is rated on a 5-point Likert scale ranging from 1 (never) to 5 (always). Total scores range from 9 to 45, with higher scores indicating better leisure crafting capabilities among nurses. In this study, the Cronbach’s *α* of the scale was 0.908.

#### 4.3.5 Stanford presenteeism scale-6

The SPS-6 was originally developed by Koopman et al. (56). In this study, the Chinese version of the SPS-6 (57) was used to measure nurses’ productivity loss due to health problems in the past month. The scale includes two dimensions (work limitation and work energy), comprising six items rated on a 5-point Likert scale ranging from 1 (completely disagree) to 5 (completely agree). Total scores range from 6 to 30, with higher scores indicating greater health-related productivity loss caused by presenteeism among nurses. In this study, the Cronbach’s *α* of the scale was 0.861.

### 4.4 Ethical declaration

This study was approved by the Ethics Committee of Chengdu Medical College (Approval no. CMCIR2025NO.001). After obtaining electronic informed consent from each nurse, the study invited all participants to complete the questionnaire anonymously. They were explicitly informed that their responses would be kept strictly confidential and that they had the right to decline participation in this study.

### 4.5 Data collection

The questionnaire was converted into an electronic format using the online platform Wenjuanxing.^1^ With the assistance of nursing directors and head nurses, the survey link and QR code were distributed to clinical nurses via WeChat work groups. Participants could access the questionnaire by clicking the link or scanning the QR code. The first page of the questionnaire contained a standardized introduction explaining the study’s purpose, significance, and instructions for completion and emphasized voluntary participation, anonymity, and informed consent. Nurses who consented to participate could proceed by clicking the “Agree” button, while those who declined consent were restricted from accessing the questionnaire. Each IP address or WeChat account could only submit the questionnaire once, to prevent the same participant from submitting multiple times. To ensure the quality of collected data, all completed questionnaires were independently reviewed and entered by two researchers, and responses with logical inconsistencies, obvious errors, or patterned anomalies were excluded. In addition, all items were set as mandatory, and participants had to answer all questions before submitting, to ensure data completeness. Before the formal survey, 20 clinical nurses who met the inclusion criteria participated in a pilot test, and the data of these participants were not included in the final analysis. The focus was on collecting their feedback on how well they understood the questionnaire items, and the completion time was recorded. The pilot test confirmed that all items were clearly understood, no modifications were necessary, and the average time for completion was approximately 10–15 min. A total of 475 questionnaires were collected. Invalid responses were excluded, and 458 valid questionnaires were retained, yielding an effective response rate of 96.42%.

### 4.6 Statistical analysis

Prior to testing the research model, Harman’s single-factor test was conducted to assess potential common method bias. Subsequently, two confirmatory factor analyses (CFAs) were conducted to evaluate the validity and reliability of the measurement model by calculating Cronbach’ s alpha coefficients, composite reliability (CR), and average variance extracted (AVE) for each scale. In addition to descriptive statistics, Pearson correlation analyses were conducted to examine correlations among variables. Referring to previous studies (58, 59), this study included all demographic variables such as gender, age, marital status, education level, professional title, self-rated health status, and years of work experience as control variables to minimize the influence of these factors on the results. Finally, hypotheses were tested using SPSS 26.0 and the PROCESS macro (Models 4 and 7). Additionally, 95% bootstrap confidence intervals (CI) were calculated based on 5,000 bootstrap samples, and the significance level for all statistical tests was set at *α* = 0.05.

## 5 Results

### 5.1 Demographic characteristics

A total of 458 nurses were included in this study, of whom 95.0% were female. Participants were predominantly aged between 26 and 35 years (48.0%), mostly married (53.3%), and primarily held bachelor’ s degrees (50.8%) or associate degrees (48.3%). Regarding professional titles, senior nurses accounted for the largest proportion (42.8%), followed by nurses (35.8%). Self-rated health status was predominantly fair (56.3%), and nearly half of the participants had 1–5 years of work experience (46.7%). Further details are presented in Table 1.

**Table 1:** General demographic information (*N* = 458).

Variable	Number	Percentage (%)
Gender		
Male	23	5.0
Female	435	95.0
Age		
<25	116	25.3
26 ~ 35	220	48.0
36 ~ 45	113	24.7
> 45	9	2.0
Marital status		
Unmarried	199	43.4
Married	244	53.3
Divorced or widowed	15	3.3
Education level		
Associate degree	221	48.3
Bachelor degree	233	50.8
Master degree	4	0.9
Professional title		
Nurse	164	35.8
Senior nurse	196	42.8
Supervisor nurse or above	98	21.4
Self-rated health status		
Poor	164	35.8
Fair	258	56.3
Good	36	7.9
Years of work experience		
1 ~ 5	214	46.7
6 ~ 10	124	27.1
11 ~ 15	83	18.1
>15	37	8.1

### 5.2 Validity and reliability of the scales

A Harman’s single-factor test was conducted to assess common method bias. The result showed that one factor accounted for 38.896% of the variance, below the 40% threshold, indicating no serious common method bias (60).

To further evaluate the structural independence of the four constructs in our model and rule out the presence of a common underlying factor, we conducted two CFAs. We compared a one-factor model, in which all items were loaded onto a single factor, and a four-factor model, in which items were grouped according to their respective constructs. As expected, the one-factor model showed poor fit (*χ*^2^/df = 3.34, CFI = 0.77, TLI = 0.76, RMSEA = 0.07 and SRMR = 0.09), while the four-factor model exhibited an excellent fit (*χ*^2^/df = 1.28, CFI = 0.97, TLI = 0.97, RMSEA = 0.02, and SRMR = 0.03), supporting the validity of our measurement model. In addition, the reliability Cronbach’s alpha, CR, AVE values reported in Table 2 confirmed the robustness of our scales.

**Table 2:** Reliability indices, descriptive statistics, and correlations among variables.

Variables	M (SD)	CR	AVE	1	2	3	4
1. Job stress	94.26 (20.24)	0.97	0.52	(0.97)			
2. Emotional exhaustion	17.34 (4.50)	0.86	0.55	0.36^**^	(0.86)		
3. Leisure crafting	26.47 (6.19)	0.91	0.52	−0.47^**^	−0.37^**^	(0.91)	
4. Presenteeism	17.42 (4.20)	0.86	0.51	0.53^**^	0.53^**^	−0.53^**^	(0.86)

Cronbach’s alphas in brackets on the diagonal. ^***^p < 0.001; ^**^p < 0.01; ^*^p < 0.05; M, Mean; SD, Standard deviation.

### 5.3 Correlation analysis

Table 2 displayed the descriptive statistics and correlation matrix for main variables. Job stress was significantly and positively correlated with emotional exhaustion (r = 0.36, *p* < 0.01) and presenteeism (*r* = 0.53, *p* < 0.01). Leisure crafting showed significant negative correlations with job stress (*r* = −0.47, *p* < 0.01), emotional exhaustion (*r* = −0.37, *p* < 0.01), and presenteeism (*r* = −0.53, *p* < 0.01). Emotional exhaustion was positively correlated with presenteeism (*r* = 0.53, *p* < 0.01).

### 5.4 Testing for mediation

Using job stress as the independent variable, presenteeism as the dependent variable, and emotional exhaustion as the mediator, mediation analysis was performed using standardized variables with PROCESS macro Model 4. All demographic variables were included as covariates in the final model. The results indicated that job stress significantly and positively predicted presenteeism (*β* = 0.524, *p* < 0.001). After including the mediator, job stress remained a significant predictor of presenteeism (*β* = 0.391, *p* < 0.001) and significantly predicted emotional exhaustion (*β* = 0.354, *p* < 0.001), which in turn predicted presenteeism (*β* = 0.377, *p* < 0.001; Table 3). The bias-corrected bootstrap test indicated that emotional exhaustion partially mediated this relationship, with an indirect effect of 0.133, 95% CI (0.099, 0.171), accounting for 25.38% of the total effect (Table 4). Thus, hypotheses 1 and 2 were supported.

**Table 3:** Mediation effects of emotional exhaustion on the relationship between job stress and presenteeism.

Variables	Model 1 Presenteeism		Model 2 Emotional exhaustion		Model 3 Presenteeism	
	*β*	*t*	*β*	*t*	*β*	*t*
Gender	0.104	0.578	0.070	0.348	0.078	0.475
Age	0.001	0.013	0.037	0.418	−0.013	−0.179
Marital status	0.093	0.970	0.015	0.136	0.088	1.005
Education level	−0.146	−1.672	−0.255	−2.606^**^	−0.050	−0.626
Professional title	0.002	0.031	−0.051	−0.680	0.021	0.351
Self-rated health status	−0.168	−2.527^*^	−0.002	−0.027	−0.167	−2.772^**^
Years of work experience	0.126	2.407^*^	0.066	1.119	0.101	2.129^*^
Job stress	0.524	13.041^***^	0.354	7.863^***^	0.391	10.043^***^
Emotional exhaustion					0.377	9.850^***^
*R* ^2^	0.319		0.146		0.440	
*F*	26.260		9.596		39.116	

^***^p < 0.001; ^**^p < 0.01; ^*^p < 0.05.

**Table 4:** The bootstrapping analysis of the mediating effects.

Effect type	Effect	Boot SE	Bootstrap 95%CI		Relative mediation effect
			BootLL CI	BootUL CI	
Total effect	0.524	0.040	0.445	0.603	100%
Direct effect	0.391	0.039	0.314	0.467	74.62%
Indirect effect	0.133	0.018	0.099	0.171	25.38%

### 5.5 Testing for moderated mediation

Using PROCESS macro Model 7 to test the moderating effect of leisure crafting. Leisure crafting was introduced into the mediation model as a moderator, while controlling for demographic variables. Results indicated that job stress significantly and positively predicted emotional exhaustion (*β* = 0.251, *p* < 0.001), whereas leisure crafting significantly and negatively predicted emotional exhaustion (*β* = −0.234, *p* < 0.001). Moreover, the interaction term between job stress and leisure crafting significantly predicted emotional exhaustion (*β* = −0.090, *p* < 0.05), with an index of moderated mediation of −0.034, SE = 0.015, 95%CI = (−0.064, −0.005), indicating that leisure crafting moderated the relationship between job stress and emotional exhaustion (Table 5).

**Table 5:** Results of leisure crafting moderated the mediation process.

Variables	Model 1 Emotional exhaustion		Model 2 Presenteeism	
*β*	*t*	*β*	*t*
Gender	0.026	0.130	0.078	0.475
Age	0.028	0.329	−0.013	−0.179
Marital status	0.010	0.096	0.088	1.005
Education level	−0.238	−2.495^*^	−0.050	−0.626
Professional title	−0.022	−0.296	0.021	0.351
Self-rated health status	0.035	0.476	−0.167	−2.772^**^
Years of work experience	0.059	1.035	0.101	2.129^*^
Job stress	0.251	5.107^***^	0.391	10.043^***^
Leisure crafting	−0.234	−4.777^***^		
Job stress×Leisure crafting	−0.090	−2.122^*^		
Emotional exhaustion			0.377	9.850^***^
*R* ^2^	0.199		0.440	
*F*	11.105		39.116	

*N* = 458. ***p < 0.001; **p < 0.01; *p < 0.05.

**Figure 2:**
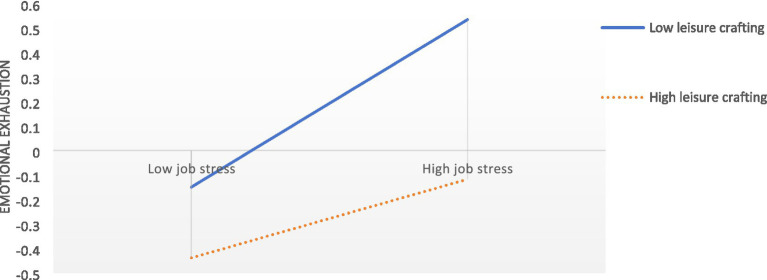
Leisure crafting as a moderator on the relationship between job stress and emotional exhaustion.

To further investigate the moderating effect of leisure crafting, simple slope analyses were conducted. Leisure crafting was categorized into high and low levels based on one standard deviation above and below the mean (see Figure 2). Results showed that job stress significantly and positively predicted emotional exhaustion at both high (*β* = 0.160, *p* < 0.05) and low (*β* = 0.341, *p* < 0.001) levels of leisure crafting, although the predictive effect was notably weaker at the high level of leisure crafting. This indicated that the higher the level of leisure crafting, the weaker the positive predictive effect of job stress on emotional exhaustion. Furthermore, bias-corrected percentile bootstrap analysis further confirmed a significant indirect effect of job stress on presenteeism via emotional exhaustion among nurses reporting high leisure crafting [*β* = 0.060, SE = 0.021, 95% CI = (0.020, 0.104)]; whereas the indirect effect was also significant but stronger among nurses reporting low leisure crafting [*β* = 0.129, SE = 0.026, 95% CI = (0.081, 0.181)] (Table 6). Therefore, hypotheses 3 and 4 were supported.

**Table 6:** Conditional indirect effect of leisure crafting when emotional exhaustion mediated between job stress and presenteeism.

Mediator	Leisure crafting	Effect	SE	Boot LLCI	Boot ULCI
Emotional exhaustion	M-1SD	0.129	0.026	0.081	0.181
	M	0.094	0.018	0.062	0.131
	M + 1SD	0.060	0.021	0.020	0.104

### 5.6 Sensitivity analyses

We conducted a sensitivity analysis by replicating all analyses without controlling for sociodemographic variables. The Supplementary Tables S1–S4 show that the results of the mediation and moderated mediation analyses did not exhibit substantial changes in significance or effect size, demonstrating the robustness of our findings.

## 6 Discussion

Grounded in the COR theory and the JD-R model, this study examined the relationship between job stress and presenteeism among Chinese nurses, emphasizing the mediating role of emotional exhaustion and the moderating role of leisure crafting. The results indicated a positive association between job stress and presenteeism. Emotional exhaustion was found to partially mediate this relationship, while leisure crafting moderated the indirect pathway from job stress to presenteeism via emotional exhaustion. These findings can offer both theoretical insights and practical implications for reducing presenteeism in nursing practice.

The results revealed a positive relationship between job stress and presenteeism among nurses, suggesting that elevated job stress directly increases the likelihood of presenteeism. Previous studies have consistently supported this relationship (17, 27). According to COR theory, job stress may be associated with nurses’ tendency to adopt presenteeism as a coping strategy to preserve resources and prevent further resource loss under high-pressure conditions (20). While this strategy may provide short-term relief, it can accelerate the depletion of physical and psychological resources over time, ultimately impairing job performance and well-being (29). Moreover, the dynamic model of absenteeism and presenteeism developed by Johns highlights job stress as a key antecedent of presenteeism behaviors (61). Accordingly, nursing administrators should proactively address job stress by implementing interventions, such as workload redistribution, optimized shift scheduling, sufficient rest periods, enhanced communication and conflict resolution mechanisms, clearer role definitions, and performance-based incentives. These strategies may help alleviate excessive stress, reduce presenteeism, and promote both nurse well-being and the quality of patient care (5).

This finding suggested that job stress increases the risk of presenteeism by intensifying emotional exhaustion. This is consistent with previous research (17, 62). It is worth noting that our findings are best interpreted within a suboptimal health framework. According to the health impairment process of the JD-R model, prolonged exposure to high job demands and stress depletes nurses’ psychological and physical resources, thereby elevating the risk of persistent emotional fatigue and eventual exhaustion (63). Emotional exhaustion is widely regarded as a core symptom of suboptimal health status, present even before the onset of diagnosable illness. When nurses experience poor health while simultaneously managing challenging stressors, they experience a loss of health-related productivity, and they ultimately engage in presenteeism (39, 64). From this perspective, emotional exhaustion constitutes both a manifestation and a precursor of suboptimal health status. The mediating pathway confirmed by this study demonstrates how sustained work stress induces a continuum of deteriorating occupational health. Therefore, nursing managers should take proactive measures to address emotional exhaustion among nurses. For instance, psychological counseling services and peer support systems can be established to provide timely emotional support (65). In addition, regular stress management training should be provided to help nurses develop effective coping strategies and relaxation techniques (66). Moreover, appropriate human resource allocation and optimized shift scheduling are essential to prevent chronic overload, ensuring adequate rest and balanced rotations to reduce the risk of emotional exhaustion.

Our study also identified that leisure crafting moderated the relationship between job stress and emotional exhaustion. While earlier studies have demonstrated a robust relationship between job stress and emotional exhaustion (67, 68), limited attention has been paid to potential buffering variables. To the best of our knowledge, this is the first empirical study to confirm the moderating effect of leisure crafting on the relationship between job stress and emotional exhaustion among clinical nurses, thus providing a novel addition to existing research. Personal resources accumulated through leisure crafting may strengthen nurses’ resource reservoirs and reduce perceived job demands, thereby mitigating emotional exhaustion associated with job stress (69). This aligns with the COR theory, which conceptualizes leisure crafting as a cross-contextual compensatory strategy that offsets stress-induced resource loss (22). Therefore, nursing managers should pay greater attention to nurses’ leisure time activities and provide necessary support. Flexible scheduling and adequate rest should be ensured to allow nurses to engage in physical exercise and pursue personal interests, thus facilitating resource recovery and stress relief. In addition, staff care and health promotion programs can be implemented to encourage nurses to develop hobbies and social networks outside of work, thereby reducing the risk of emotional exhaustion.

The final moderated mediation model provided further evidence that job stress exerts a significant indirect effect on presenteeism through emotional exhaustion, with this pathway moderated by leisure crafting. Specifically, high levels of job stress elevated emotional exhaustion among nurses, which in turn raised the likelihood of presenteeism. However, this indirect effect was weaker among those with higher levels of leisure crafting and was accompanied by lower tendencies toward presenteeism. In other words, leisure crafting equips nurses with additional psychological and physical resources, enabling them to maintain functional well-being under stress and avoid the dilemma of being physically present but mentally disengaged due to emotional exhaustion (70).

## 7 Implications

The findings of this study enhance our understanding of factors associated with nurse presenteeism, offering evidence-based guidance for developing targeted intervention strategies. For nursing administrators, reducing job stress through equitable workload distribution and optimized shift scheduling is essential to ensure adequate rest for nurses amid demanding schedules. Leadership should also provide emotional regulation support by offering regular training in stress management and emotional resilience to enhance nurses’ coping capacities. Managers are encouraged to promote leisure crafting by providing recreational activities, access to fitness facilities, and flexible leave policies, while respecting nurses’ non-work time and encouraging participation in meaningful off-duty pursuits. For individual nurses, raising awareness of emotional regulation and proactively utilizing organizational training and resources are essential for enhancing emotional coping. Techniques such as deep breathing, cognitive reframing, and timely support-seeking from peers and supervisors (71, 72) can help nurses effectively manage negative emotions in the workplace. In addition, nurses should strive to maintain a healthy work–life balance by actively engaging in physical activities, personal interests, and leisure pursuits outside of working hours to promote both psychological and physical well-being.

## 8 Limitations and future research

Although this study enriched both the theoretical and empirical understanding of the relationship between job stress and presenteeism among nurses, several limitations should be acknowledged. First, the study employed a cross-sectional design, in which all variables were collected at the same time point, making it difficult to determine the direction of causality among them. Future research should consider longitudinal or time-lagged designs to more robustly test causal mechanisms.

Second, all variables in this study were measured via nurses’ self-reports, leading to single-source data, which may introduce response bias and affect the accuracy of the findings. It is recommended that future studies collect multi-source data, such as peer ratings, supervisor evaluations, and assessments from medical and administrative staff, to reduce bias from subjective reports and improve measurement objectivity (60, 73).

Third, this study employed convenience sampling, and the sample was drawn from three tertiary hospitals in Sichuan Province, which may limit the overall representativeness of the sample, and may introduce potential urban–rural bias. The study conclusions should be cautiously generalized to primary healthcare institutions and rural areas. Accordingly, future research should consider adopting multi-center and stratified sampling strategies, incorporating hospitals from diverse regions and different healthcare levels, to enhance sample diversity and improve the external validity of the findings.

Fourth, the study sample consisted predominantly of female nurses (95%) and relatively young participants (73.3%). Although this structure is generally consistent with the national nursing workforce (74), it may still limit the external validity of the results. Future studies should include more male and older nurses to verify the generalizability of the findings in broader populations.

Finally, this study may not have fully accounted for certain key mechanisms. For example, professional identity and psychological capital, as important internal psychological resources, may mediate the relationship between job stress and presenteeism (75, 76). Future studies could include these variables to further enrich relevant theoretical models and provide empirical evidence for developing targeted organizational intervention strategies.

## 9 Conclusion

This study used Chinese nurses as the sample to explore the mechanism by which job stress is associated with presenteeism through emotional exhaustion, and examined the moderating effect of leisure crafting on the relationship between job stress and emotional exhaustion. Job stress depletes nurses’ emotional reserves, increasing their risk of transitioning from a healthy status to a suboptimal health status characterized by emotional exhaustion, thereby heightening the likelihood of presenteeism. Simultaneously, presenteeism manifests as behavioral evidence of this latent health impairment, placing nurses along the continuum of occupational health deterioration. Leisure crafting has a significant buffering effect on the relationship between job stress and emotional exhaustion: the higher the level of leisure crafting, the lower the degree of emotional exhaustion caused by job stress. These findings highlight the need for nurse managers and policymakers to prioritize job stress and emotional exhaustion as early targets for occupational health interventions. This approach may help prevent nurses from progressing along the continuum of deteriorating occupational health and reduce the likelihood of presenteeism.

## Data Availability

The raw data supporting the conclusions of this article will be made available by the authors, without undue reservation.

## Footnotes

1
https://www.wjx.cn/
