# Supplementary material for: Evaluating the association between job stress and presenteeism among nurses: the mediating role of emotional exhaustion and the moderating effect of leisure crafting
Source: Front Public Health. 2026 Jan 26;14:1719915. doi: 10.3389/fpubh.2026.1719915 (PMC12883798; doi:10.3389/fpubh.2026.1719915)
Supplement: Supplementary file 1 [file Supplementary_file_1.docx]

**Supplementary materials**

**Table S1** Mediation effects of emotional exhaustion on the relationship between career calling and presenteeism.

| **Variables** | **Model 1**  **Presenteeism** | | **Model 2**  **Emotional exhaustion** | | **Model 3**  **Presenteeism** | |
| --- | --- | --- | --- | --- | --- | --- |
|  | **β** | **t** | **β** | **t** | **β** | **t** |
| Job stress | 0.533 | 13.448^***^ | 0.358 | 8.184^***^ | 0.395 | 10.276^***^ |
| Emotional exhaustion |  |  |  |  | 0.384 | 9.986^***^ |
| R^2^ | 0.284 | | 0.128 | | 0.413 | |
| F | 180.843 | | 66.985 | | 159.858 | |
| ^**^*^*^p*<0.001; ^**^*p*<0.01; ^*^*p*<0.05 | | | | | | |

**Table S2** The Bootstrapping analysis of the mediating effects.

|  | **Effect** | **Boot SE** | **Bootstrap 95%CI** | **Relative mediation effect** |
| --- | --- | --- | --- | --- |
|  |  |  | **BootLL CI BootUL CI** |  |
| Total effect | 0.533 | 0.040 | 0.455 0.611 | 100% |
| Direct effect | 0.395 | 0.038 | 0.320 0.471 | 74.11% |
| Indirect effect | 0.138 | 0.018 | 0.105 0.173 | 25.89% |

**Table S3** Results of leisure crafting moderate the mediation process.

| **Variables** | **Model 1**  **Emotional exhaustion** | | **Model 2**  **Presenteeism** | |
| --- | --- | --- | --- | --- |
|  | **β** | **t** | **β** | **t** |
| Job stress | 0.245 | 5.090^***^ | 0.395 | 10.276^***^ |
| Leisure crafting | -0.245 | -5.093^***^ |  |  |
| Job sress×Leisure crafting | -0.084 | -1.997^*^ |  |  |
| Emotional exhaustion |  |  | 0.384 | 9.986^***^ |
| R^2^ | 0.185 | | 0.413 | |
| F | 35.350 | | 159.858 | |
| N=458. ^**^*^*^p*<0.001; ^**^*p*<0.01; ^*^*p*<0.05 | | | | |

**Table S4** Conditional indirect effect of leisure crafting when emotional exhaustion mediated between job stress and presenteeism.

| **Mediator** | **Leisure crafting** | **Effect** | **SE** | **Boot LLCI** | **Boot ULCI** |
| --- | --- | --- | --- | --- | --- |
| Emotional exhaustion | M-1SD | 0.126 | 0.024 | 0.081 | 0.177 |
|  | M | 0.094 | 0.017 | 0.063 | 0.129 |
|  | M + 1SD | 0.062 | 0.021 | 0.022 | 0.102 |
| Index of moderated mediation |  | -0.032 | 0.015 | -0.062 | -0.05 |
